# Effects of a home-based intervention on diet and physical activity behaviours for rural adults with or at risk of metabolic syndrome: a randomised controlled trial

**DOI:** 10.1186/s12966-016-0337-2

**Published:** 2016-02-01

**Authors:** Krysten Blackford, Jonine Jancey, Andy H. Lee, Anthony James, Peter Howat, Tracy Waddell

**Affiliations:** Collaboration for Evidence, Research and Impact in Public Health (CERIPH), Curtin University, Perth, WA Australia; School of Public Health, Curtin University, Perth, WA Australia

**Keywords:** IPAQ-SF, Strength exercise, Walking, Sitting, Fibre intake, Fruit and vegetable intake, Fat avoidance, Obesity, Metabolic syndrome, Disadvantage

## Abstract

**Background:**

This study aimed to determine whether a home-based 6-month lifestyle intervention program complemented by motivational interviewing could improve diet and physical activity behaviours in 50–69 year olds with or at risk of metabolic syndrome, residing in a disadvantaged rural Western Australian community.

**Methods:**

Participants from the City of Albany and surrounding towns (*n* = 401) were recruited into a 6 month randomised controlled trial. They were screened for metabolic syndrome and randomly allocated to intervention (*n* = 201) or control group (*n* = 200). Baseline and post-test data collection for both groups included a self-report questionnaire which incorporated the Fat and Fibre Barometer and the International Physical Activity Questionnaire Short Form. The intervention group received the program materials at baseline and the control group was waitlisted. Generalised estimating equation models assessed repeated outcome measures over time.

**Results:**

A total of 151 (75.1 %) intervention and 159 (79.5 %) control group participants completed post-test and were included in the analysis. After controlling for confounders, the intervention group achieved a marginally significant increase in their metabolic equivalent (MET) minutes of moderate intensity physical activity per week (*p* = 0.049), and significantly improved fibre intake (*p* < 0.001), fat intake (*p* = 0.003), and vegetable serves per day (*p* = 0.002) from baseline to post-test relative to the control group.

**Conclusion:**

A home-based, low-cost intervention with motivational support can effectively improve the physical activity and dietary behaviours of adults aged 50–69 years with or at risk of metabolic syndrome residing in a disadvantaged rural area.

**Trial registration:**

Anzctr.org.au Identifier: ACTRN12614000512628

## Background

Metabolic syndrome is characterised by several metabolic abnormalities including raised triglyceride levels, reduced high density lipoprotein (HDL) cholesterol, hypertension, hyperglycaemia, and abdominal obesity [1]. Individuals with metabolic syndrome are at a significantly increased risk of developing chronic diseases such as type 2 diabetes and cardiovascular disease [1, 2]. Prevalence of metabolic syndrome varies between populations, with estimates of approximately 13-30 % of adults in developing countries and 35 % in developed countries such as Australia and the USA [3]. Data from the United Kingdom suggests that prevalence increases with age [3].

Several risk factors are responsible for the majority of metabolic syndrome cases and cardiovascular diseases in developed countries [4]. These include high cholesterol, overweight/obesity, physical inactivity, high blood pressure, and limited fruit and vegetable intake [4]. The risk factors contributing significantly to the burden of disease in Australia are poor diet (11 %) and overweight/obesity (9 %) [5]. In 2011–12, 92 % of Australian adults were not consuming enough serves of vegetables to meet the Australian Dietary Guidelines, and just 49 % achieved the recommended target for fruit consumption [5], while only 40 % met the recommendation of at least 150 min per week of moderate intensity physical activity [5].

The gap in health risk behaviours between the least and most disadvantaged groups of the Australian population is widening, particularly for fruit and vegetable intake [6]. People living in lower socioeconomic areas of Australia are more likely to be physically inactive, engage in sedentary behaviour, and have abnormal glucose metabolism compared to those living in higher socioeconomic areas [7]. Similar effects have been noted across seven comparable countries, with higher socioeconomic neighbourhoods associated with increased consumption of fruit and vegetables [8].

Residents of rural and remote Australia experience higher rates of morbidity and mortality and have less access to health services than those in metropolitan areas [5]. These populations are more likely than city populations to be overweight or obese (70 versus 60 %), insufficiently active (60 versus 54 %), have high blood cholesterol (37 versus 31 %) and comprise more people aged over 65 years (16 versus 13 %) [5]. This results in an increased prevalence of overweight/obesity, metabolic syndrome, and in turn chronic diseases [9]. Targeted screening and interventions in these populations at high risk of developing cardiovascular disease and type 2 diabetes may lead to early identification of metabolic syndrome, early management and delayed onset of these chronic diseases [10, 11].

Interventions to address metabolic syndrome and related chronic diseases in older adults should encourage reduced sitting time and increased physical activity [12] in combination with diet modifications. These include limiting saturated fat, sugar and salt, and increasing fibre, fruit and vegetable consumption [1, 13, 14]. The literature indicates that structured behavioural interventions focusing on counselling, education, and support strategies can also assist positive behaviour change in individuals at risk of chronic diseases [15].

A recent systematic review of self-help interventions for adults at risk of chronic diseases suggests that strategies comprising goal setting and self-monitoring in combination with tailored feedback, contact via email, and online social support, may be more effective for achieving behaviour change [16]. However, it is difficult to determine the effectiveness of self-help interventions in disadvantaged target groups due to insufficient data [16]. In particular, there is a gap in the knowledge of the impact of home-based lifestyle interventions in the disadvantaged rural/remote Australian setting targeting older adults with metabolic syndrome [17].

The Albany Physical Activity and Nutrition (APAN) Program aimed to improve dietary and physical activity behaviours of 50–69 year old adults at risk or with metabolic syndrome. The APAN program was implemented in the City of Albany and surrounding towns in The Great Southern region of Western Australia, which provided access to a large number of older adults aged 50–69 years (8496) [18]. The Socio-Economic Indexes for Areas scores Albany at 987.4, indicating relative disadvantage (<1000) [19]. This population is representative of other Australian rural/regional areas. In addition, the Great Southern region’s health profile suggests a need for increased access to health programs and services for residents [20]. The present study aimed to determine whether the APAN program effectively improved the physical activity, diet, and sedentary behaviours of participants.

## Methods

### Study design

The protocol of this trial has been described previously [21]. APAN was a two-arm randomised controlled trial of a 6-month physical activity, diet, and healthy weight management intervention. Data were collected from the intervention and control groups at baseline and post-test. The trial was registered with the Australian and New Zealand Clinical Trials Registry (ACTRN12614000512628) and the study protocol was approved by the Curtin University Human Research Ethics Committee (approval number HR149_2013). All participants provided informed consent prior to entry into the study.

### Participants

Potential participants were required to be 50–69 years of age and classified as either at risk of, or with metabolic syndrome using the International Diabetes Federation (IDF) criteria [22] to be eligible for the study. To be considered at risk, participants were required to have a large waist circumference as the minimum requirement (waist circumference ≥94 cm for men or ≥80 cm for women [Europids, Sub-Saharan Africans, Eastern Mediterranean, Middle East]; ≥90 cm for men or ≥80 cm for women [South Asians, Chinese, Japanese]), plus one of the following parameters: raised triglyceride concentration (≥1.7 mM, or treatment for this); reduced HDL-cholesterol concentration (<1.03 mM in males and <1.29 mM in females, or treatment for this); raised blood pressure (systolic ≥130 mmHg or diastolic ≥85 mmHg, or treatment of previously diagnosed hypertension); raised fasting plasma glucose concentration (≥5.6 mM). Participants were classified as having metabolic syndrome if they had a large waist circumference plus two of the other criteria stated above [23].

The following exclusion criteria applied: on a weight loss diet or having weight fluctuations of >5 % within the previous 6 months; previous diagnosis of diabetes mellitus (other than gestational diabetes); of Aboriginal or Torres Strait Islander decent; receiving specific treatment to lower blood glucose; or involvement in another physical activity program.

### Procedure

**Fig. 1:**
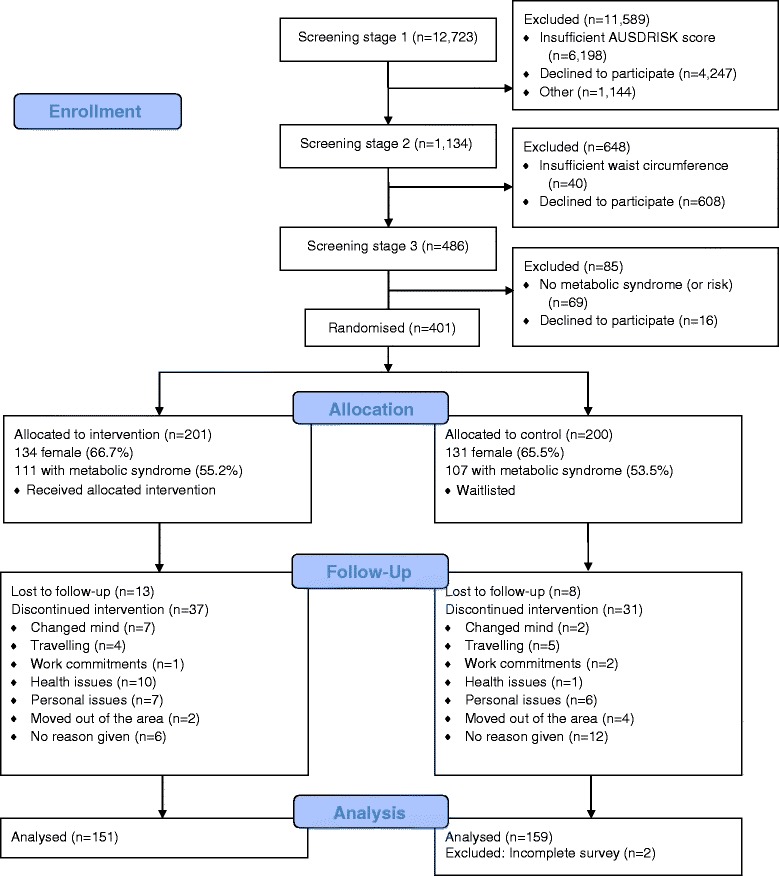
Consort flow diagram

Participants aged 50–69 years were recruited from towns within a 50 km radius of Albany, Western Australia. Recruitment of participants occurred in three stages. Figure 1 outlines the study procedure, participant flow, and samples sizes.

#### Screening stage 1

Individuals (*n* = 12,723) from the selected region were initially screened via the Computer Assisted Telephone Interview system using the Australian Type 2 Diabetes Risk Assessment Tool (AUSDRISK), which assesses the risk of developing type 2 diabetes mellitus based on anthropometric, demographic, and lifestyle characteristics [24]. Females with a score of ≥ 9 and males with a score of ≥ 12 were eligible for the next stage of screening (*n* = 1134), with 7342 individuals excluded and 4247 individuals who opted out.

#### Screening stage 2

Eligible individuals (*n* = 1134) were invited to attend a clinic in a central location in Albany. A total of 526 participants attended the clinic and 608 opted out. Anthropometry including waist circumference was measured during the appointment to confirm central obesity (waist circumference ≥94 cm for men or ≥80 cm for women, being the minimum IDF requirement) before progressing to the next stage. Based on this eligibility criterion, 40 participants were excluded, and a further 16 opted out after progressing to screening stage 3.

#### Screening stage 3

The remaining eligibility criteria were assessed at a local pathology laboratory via fasting blood sample and blood pressure measurements. Participants also completed a self-reported questionnaire to measure physical activity and dietary behaviours.

Participants meeting the full eligibility criteria (*n* = 401) were randomly allocated to either the intervention group (*n* = 201) or control group (*n* = 200), while adjusting for gender and metabolic syndrome status. Intervention group participants were provided the APAN program materials and allocated to one of two motivational support staff. The control group participants were waitlisted to receive their program after post-test data collection (6 months).

Baseline and post-test data collection included a self-reported questionnaire to measure participant dietary and physical activity behaviours. Of the 201 intervention and 200 control group participants who completed the questionnaire at baseline, 151 (75.1 % response rate) and 159 (79.5 % response rate) respectively completed the post-test questionnaire and were available for analysis of the self-reported outcome measures.

### Intervention

This home-based program was low cost and tailorable to suit varying fitness levels and lifestyles. Its strategies included self-monitoring and goal setting, supported by printed and online material, and participants received email and telephone support using motivational interviewing techniques. The program built upon previous dietary and physical activity programs targeting older adults in a metropolitan area [25, 26], and was adapted for a rural/remote target group.

#### Theoretical basis

The Self-Determination Theory was applied as the theoretical framework for this study, complemented by Motivational Interviewing [27]. The theory explores human motivation in terms of behaviours that are autonomous (originating from self) and behaviours that are controlled (persuasion or coercion) [28]. Self-Determination Theory is the only motivational theory that identifies autonomy as a basic need for all humans that should be supported in health promotion interventions [29]. Individuals are more likely to engage in certain behaviours if they are valued and intrinsically motivated [30]. Advice from health professionals should be offered in an autonomous manner to allow for individual decision-making [27].

#### Program materials

Intervention group participants were provided with the APAN program materials designed to assist with self-monitoring and goal setting to improve diet, physical activity, and healthy weight maintenance. Recommendations were based on Australia’s Physical Activity and Sedentary Behaviour Guidelines [31] and the Australian Dietary Guidelines [32], as well as previous successful interventions targeting older adults [25, 26]. Materials included a booklet, exercise charts, resistance band, nutrition panel wallet card, and a website with progress tracker and interactive blog. A detailed overview of the program materials has been described previously [21].

#### Motivational support

Intervention group participants were provided motivational support for the duration of the APAN intervention via telephone counselling and emails. Two research assistants trained in motivational interviewing techniques contacted participants at week 1, 3, 6, 12, 18, and 24. Motivational interviewing principles incorporate strategies to encourage an individual to initiate and sustain behaviour change, such as empathy, shared decision-making, and reflective listening [33]. These strategies complemented the principles of the Self-Determination Theory by ensuring participants set their own goals, became involved in decision-making, and were not coerced into a particular behaviour or action [27, 30].

Research assistants were provided with a dialogue guide for each of the six MI telephone calls scheduled over the intervention period. The first call focused on general health as the main topic, allowing the research assistants to introduce themselves to participants and gauge their readiness to change, barriers to change, and identify specific goals they wished to achieve. Subsequent calls focused on specific topics including physical activity, sedentary behaviour, nutrition, and the revisiting of goals. Participants were encouraged to utilise their APAN resources to monitor their progress and adjust goals and behaviours as required.

### Instrument

The self-completed structured questionnaire included the Fat and Fibre Barometer [34], the International Physical Activity Questionnaire Short Form (IPAQ-SF) [35], and demographic and general health questions including sex, education, marital status, smoking status, diagnosed health conditions, medications, and alcohol consumption. The IPAQ-SF measured walking time, moderate and vigorous intensity physical activities, and time spent sitting across a usual week. A strength question was also added to determine resistance or weight training using large muscle groups [26].

To assess diet, the Fat and Fibre Barometer was utilised to provide information on habitual fat- and fibre-related behaviours of participants [34]. The valid and reliable instrument was appended with questions asking participants for the number of fruit and vegetable serves in a usual week [26]. Body mass index (BMI) was calculated based on weight and height data collected during screening stage 2.

### Statistical analysis

This paper focuses on dietary and physical activity behaviours whereas changes in clinical metabolic syndrome parameters and anthropometry will be reported elsewhere. Descriptive statistics summarised the baseline lifestyle and demographic characteristics of the intervention and control groups. Independent and paired samples t-tests were applied to the continuous outcome variables, whereas Mann-Whitney U test and Wilcoxon Signed Rank test were applied to those variables exhibiting skewed distributions. To account for the effects of potential confounders, generalised estimating equation (GEE) models with exchangeable correlation structure were used to assess the repeated outcome variables over time. Normal GEE with identity link was applied to normally distributed continuous outcome variables (sitting time [hours per day]; fibre intake score; fat intake score; fat avoidance score), while gamma GEE with log link was applied to skewed continuous variables (walking time [MET min/week]; moderate intensity activity [MET min/week]; vigorous intensity activity [MET min/week]; total activity [MET min/week]; strength training [min/week]; fruit intake [serves per day]; vegetable intake [serves per day]). All statistical analyses were performed using the SPSS Statistics Package 22.

## Results

Demographic and lifestyle characteristics of participants are summarised in Table 1, which highlight no significant differences between the intervention and control groups at baseline. The mean age of the sample was 61 years with 66.5 % female participants. The majority (98.4 %) had completed either secondary school or tertiary education, and 81.6 % had a partner. The mean BMI was 30.8 indicating that on average, the sample was obese. Smoking status and alcohol consumption were not significantly different between the two groups.

**Table 1:** Baseline characteristics of intervention and control group participants

Variable	Intervention group	Control group	*p* value^a^
	(*n* = 151)	(*n* = 159)	
Age: mean (SD) years	60.5 (5.64)	61.3 (5.18)	0.181
Metabolic syndrome status:			0.494
With	79 (52.3 %)	90 (56.6 %)	
At risk	72 (47.7 %)	69 (43.4 %)	
BMI: mean (SD)	31.0 (5.88)	30.6 (5.17)	0.440
Gender: female	100 (66.2 %)	106 (66.7 %)	0.934
Employment status:			0.296
Full time	78 (51.7 %)	65 (40.9 %)	
Part time	24 (15.9 %)	29 (18.2 %)	
Unemployed	5 (3.3 %)	7 (4.4 %)	
Retired	44 (29.1 %)	58 (36.5 %)	
Education:			0.425
Primary school	3 (2.0 %)	2 (1.3 %)	
Secondary school	55 (36.4 %)	72 (45.0 %)	
Technical/Diploma	52 (34.4 %)	46 (28.8 %)	
University	41 (27.2 %)	39 (24.5 %)	
Relationship status: with partner	124 (82.1 %)	129 (81.1 %)	0.810
Smoking status:			0.852
Never	84 (55.6 %)	84 (52.8 %)	
Ex-smoker	52 (34.4 %)	54 (33.8 %)	
Occasional smoker	3 (2.0 %)	4 (2.5 %)	
Daily smoker	12 (7.9 %)	17 (10.6 %)	
Co-morbidity^b^: yes	92 (60.9 %)	104 (65.4 %)	0.413
Alcohol drinking: yes	99 (65.6 %)	113 (71.1 %)	0.957

^a^ *t*-test or chi square test between intervention and control groups

^b^ Presence of at least one of 8 common health problems

### Physical activity outcomes

The self-reported physical activity outcomes between intervention and control groups are displayed in Table 2. Significant improvements in mean walking time (MET min/week), moderate intensity activity (MET min/week), total activity (MET min/week), sitting time (hours per day), and strength training (minutes per week) from baseline to post-test were observed for the intervention group, while a significant reduction (17 min/week) in total MET min/week was observed for the control group from baseline to post-test. Table 3 provides the results of the GEE analyses for physical activity outcomes. After controlling for potential confounders, the intervention group achieved a marginally significant improvement in the self-reported moderate intensity MET minutes per week (*p* = 0.049) relative to the control group. No significant improvement was observed for the other physical activity variables through the group x time interaction term.

**Table 2:** Comparison of self-reported physical activity outcomes between intervention and control groups

Outcome	Intervention group (*n* = 151)		p value^a^	Control group (*n* = 159)		*p* value^b^	*p* value^c^	*p* value^d^
	Baseline	Post		Baseline	Post			
	Mean (SD)	Mean (SD)		Mean (SD)	Mean (SD)			
Walking MET min/week^e^	396.0	577.5	0.011	330.0	396.0	0.047	0.561	0.050
	(561.0)	(742.5)		(594.0)	(709.0)			
Moderate MET min/week^e^	300.0	480.0	<0.001	360.0	360.0	0.808	0.394	0.189
	(585.0)	(850.0)		(640.0)	(636.0)			
Vigorous MET min/week^e^	181.5	217.5	0.070	203.0	179.5	0.715	0.902	0.176
	(479.4)	(460.2)		(462.1)	(428.4)			
Total MET min/week^e^	807.5	1332.0	<0.001	990.0	973.0	0.013	0.428	0.051
	(1486.9)	(1624.9)		(1357.5)	(1738.0)			
Strength min/week^e^	39.2	53.9	<0.001	27.7	25.0	0.436	0.880	0.001
	(168.9)	(124.2)		(66.8)	(70.4)			
Sitting hours/day	359.1	319.7	0.001	356.0	339.4	0.187	0.955	0.299
	(187.4)	(162.7)		(171.3)	(169.4)			

^a^ Paired t-test (Wilcoxon signed-rank test) between baseline and post-test for the intervention group

^b^ Paired t-test (Wilcoxon signed-rank test) between baseline and post-test for the control group

^c^ Independent t-test (Mann-Whitney *U* test) between intervention and control group at baseline

^d^ Independent *t*-test (Mann-Whitney U test) between intervention and control group at post-test

^e^Non-parametric tests applied

**Table 3:** Regression analysis of physical activity outcomes before and after intervention (*n* = 310)

	Group: intervention		Time: post		Group x time	
	Coefficient (SE)	p^c^	Coefficient (SE)	p^c^	Coefficient (SE)	p^c^
Walking MET min/wk^a^	0.09 (0.16)	0.580	0.23 (0.10)	0.020	−0.10 (0.16)	0.524
Moderate MET min/wk^a^	−0.07 (0.16)	0.650	0.01 (0.11)	0.991	0.29 (0.15)	0.049
Vigorous MET min/wk^a^	−0.21 (0.19)	0.275	−0.14 (0.17)	0.393	0.15 (0.24)	0.537
Total MET min/wk^a^	−0.05 (0.13)	0.710	0.12 (0.08)	0.164	0.11 (0.12)	0.335
Strength min/wk^a^	0.01 (0.32)	0.983	−0.40 (0.22)	0.066	0.15 (0.34)	0.653
Sitting hours/day^b^	−10.61 (19.71)	0.590	−18.10 (12.54)	0.149	−20.86 (17.16)	0.224

^a^Gamma generalised estimating equation model with log link

^b^Normal generalised estimating equation model with identity link

^c^Adjusted for age, gender, relationship status, education level, employment status, co-morbidity, alcohol drinking, and smoking status

### Dietary outcomes

The dietary outcomes are compared between groups in Table 4. Significant improvements in all self-reported outcome measures from baseline to post-test were observed for the intervention group, whereas the control group demonstrated no significant changes. Significant differences were observed between groups at post-test for the fibre intake score (*p* = 0.004), fat intake score (*p* < 0.001), fruit intake (*p* = 0.001), and vegetable intake (*p* < 0.001). Table 5 provides the results of the GEE analyses for dietary outcome variables. After controlling for potential confounders, the intervention group demonstrated significant improvements in the self-reported fibre intake (*p* < 0.001), fat intake (*p* = 0.003), and serves of vegetables per day (*p* = 0.002) using the group x time interaction term.

**Table 4:** Comparison of self-reported dietary outcomes between intervention and control groups

Outcome	Intervention group (*n* = 151)		*p* value^a^	Control group (*n* = 159)		*p* value^b^	*p* value^c^	*p* value^d^
	Baseline	Post		Baseline	Post			
	Mean (SD)	Mean (SD)		Mean (SD)	Mean (SD)			
Fibre intake score	23.3 (4.2)	24.9 (4.1)	<0.001	23.4 (4.0)	23.6 (3.8)	0.313	0.852	0.004
Fat avoidance score	12.8 (3.8)	13.3 (3.8)	0.016	12.8 (3.8)	12.8 (4.0)	0.743	0.933	0.234
Fat intake score	30.9 (4.0)	32.3 (4.0)	<0.001	30.4 (3.8)	30.8 (3.4)	0.082	0.194	<0.001
Fruit intake (serves/day)^e^	1.5 (1.3)	2.0 (1.4)	<0.001	1.5 (1.2)	1.4 (1.1)	0.378	0.619	0.001
Vegetable intake (serves/day)^e^	3.0 (2.3)	3.4 (1.9)	<0.001	2.5 (2.2)	3.0 (2.3)	0.570	0.277	<0.001

^a^ Paired *t*-test (Wilcoxon signed-rank test) between baseline and post-test for the intervention group

^b^ Paired *t*-test (Wilcoxon signed-rank test) between baseline and post-test for the control group

^c^ Independent *t*-test (Mann-Whitney *U* test) between intervention and control group at baseline

^d^ Independent *t*-test (Mann-Whitney *U* test) between intervention and control group at post-test

^e^Non-parametric tests applied

**Table 5:** Regression analysis of dietary outcomes before and after intervention (*n* = 310)

	Group: intervention		Time: post		Group x time	
	Coefficient (SE)	p^c^	Coefficient (SE)	p^c^	Coefficient (SE)	p^c^
Fibre intake score^b^	−0.06 (0.45)	0.897	0.20 (0.20)	0.329	1.39 (0.32)	<0.001
Fat intake score^b^	0.57 (0.43)	0.188	0.41 (0.23)	0.071	0.97 (0.33)	0.003
Fat avoidance score^b^	0.03 (0.42)	0.942	0.05 (0.18)	0.797	0.48 (0.28)	0.086
Fruit intake^a^	0.07 (0.07)	0.327	0.04 (0.04)	0.343	0.42 (0.26)	0.106
Vegetable intake^a^	0.07 (0.06)	0.292	0.04 (0.04)	0.321	0.17 (0.06)	0.002

^a^Gamma generalised estimating equation model with log link

^b^Normal generalised estimating equation model with identity link

^c^Adjusted for age, gender, relationship status, education level, employment status, co-morbidity, alcohol drinking, and smoking status

## Discussion

Poor diet, physical inactivity, sedentary behaviour, and overweight/obesity are some of the major risk factors contributing to Australia’s burden of disease [5]. These risk factors are more prevalent in disadvantaged and rural/remote communities in the older age groups [7], with the gap widening particularly for fruit and vegetable intake [6]. Consequently, interventions targeting this high risk group are essential to address the rising prevalence of obesity, metabolic syndrome, and chronic diseases in Australia [36, 37]. Assessment of changes to diet and physical activity behaviours of study participants provides an understanding of intervention compliance and the implication of changes in outcome measures [38].

This study examined the effectiveness of the APAN program for improving the dietary and physical activity behaviours of 50–69 year old adults at with, or at risk of metabolic syndrome in a disadvantaged rural area. The sample sizes provided sufficient statistical power to evaluate the repeated measures [21]. As expected, the attrition rate was higher for the intervention group (24.9 %) than the control group (20.5 %), and comparable to similar studies [39, 40].

The APAN program utilised the AUSDRISK [24] to initially screen individuals for risk of metabolic syndrome based on their risk of developing type 2 diabetes. To date no other studies report using this screening tool to identify metabolic syndrome in large populations. The screening process identified 1060 high risk individuals for developing type 2 diabetes, of which 215 (20 %) were confirmed to have metabolic syndrome and 186 (18 %) at risk of metabolic syndrome using the IDF criteria (central obesity plus one instead of two of the additional parameters). Targeted screening using AUSDRISK and the IDF metabolic syndrome criteria allows for evaluation of a timely lifestyle intervention to prevent the onset of cardiovascular disease and type 2 diabetes in rural Australian communities.

It is recommended that lifestyle interventions for disadvantaged groups incorporate self-help strategies due to the potentially high reach and low cost of implementation [16]. A systematic review of optimal methods and strategies for achieving lifestyle behaviour change in individuals with metabolic syndrome reported that interventions incorporating motivational feedback/interviewing in combination with internet monitoring and regular personal feedback are likely to achieve the best results [41]. Additionally, telephone-based services are able to reach geographically and socially disadvantaged areas, which commonly have higher risk of chronic diseases [42]. The APAN program incorporated a combination of these strategies to ensure the program was delivered in a cost-effective manner to the relatively disadvantaged participants in a rural community [19]. The observed changes to diet (fat, fibre and vegetable intake) and physical activity behaviours (moderate intensity activity) for the intervention group suggest that this combination of strategies is effective for the high-risk target group.

The APAN program was based on the Australian Dietary Guidelines [32]. Participants were encouraged to consume a diet high in fruit and vegetables with an emphasis on fibre intake, which is the recommendation for individuals with or at risk of metabolic syndrome [43]. The significant improvement in vegetable consumption and fat and fibre intake for the intervention group suggests that the APAN program demonstrates protective dietary factors to address metabolic syndrome in the target group.

The APAN program provided strategies for participants to identify and overcome barriers to improving physical activity and diet. Identified barriers to fruit and vegetable intake in the literature include a perception that enough serves of fruit and vegetables were consumed, as well as lack of variety, difficulty changing habits, lack of time to prepare, and low quality produce [44]. It has been suggested that interventions should provide practical solutions to address these barriers, and devise strategies to effectively communicate the recommended serves of fruit and vegetables to participants [44]. The APAN program supported participants by providing detailed information on food groups, recommended serves per day, sample meal plans, and tips for incorporating more fruit and vegetables into their daily meals, and the motivational interviewing component of the intervention encouraged participants to utilise these tools on a regular basis. Therefore, the significant increase in serves of vegetables per day for the intervention group is a successful achievement.

Based on Australia’s Physical Activity and Sedentary Behaviour Guidelines [31], the APAN program recommended that participants aim for at least 150 min per week of moderate intensity physical activity in combination with a reduction in sitting time and strength training, as these activities all impact on metabolic syndrome outcomes. There was a marginal improvement in moderate intensity activity, which is encouraging as the risk of metabolic syndrome is almost doubled among adults who engage in no moderate intensity activity [43]. Although the intervention group demonstrated some improvement in sitting time, this change was not significantly different relative to the controls. Further research into strategies to encourage a reduction in sitting time in the home environment is recommended for the target group, including optimal motivational interviewing techniques to achieve this.

Loss of muscle mass and strength, or sarcopenia, affects wellbeing, physical movement, glycaemic control and blood pressure [45, 46]. Strength training is therefore a crucial component of any physical activity intervention targeting older adults due to its physiological effects [46]. The prevalence of strength training in Australian adults remains low, particularly in regional settings (21.9 % for men and 17.5 % for women) and adults aged 55+ (7 %) [46]. The intervention group did not achieve a significant increase in strength training time compared to the control group, highlighting the need for more effort to be placed on this. The APAN exercise chart was adapted from a previous study targeting older adults (60+ years) which might have been too simplistic and not challenging enough for the younger target group in the current study. Further adaptations should be considered for future interventions.

### Limitations

A limitation of this study is the use of self-reported outcome measures which introduces reporting bias; however, any inaccuracies in reporting are anticipated to be the same across intervention and control groups. Additionally, allocation concealment ensured that the participants were unaware of their group allocation which reduced the effect of any differential over-reporting of desired behaviours and under-reporting of undesired behaviours. Another limitation is the short duration of the intervention (6 months). It is commonly accepted that exposing older adults to a sufficient dose of an intervention to ensure behaviour change is maintained in the long term can be challenging [47]. Risk factors for metabolic syndrome are effectively controlled by intensive, short-term programs for weight loss; however once the programs end there are high rates of recidivism and individuals have a tendency to regain weight [41]. A follow-up study is therefore recommended to determine long-term effectiveness and sustainability of the APAN program.

## Conclusions

Rural and remote communities are often neglected in lifestyle intervention research. It is therefore imperative that future interventions focus on sustainable health outcomes in real-world settings, particularly in disadvantaged groups. Considering the increased prevalence of preventable disease risk factors, it is essential to address poor diet, physical inactivity, and sedentary behaviour to reduce the prevalence of metabolic syndrome and related chronic diseases. The APAN program improved the physical activity and diet of the intervention group compared to the control group, demonstrating that a home-based, low-cost intervention with motivational support can effectively influence certain behaviour change of 50–69 year old adults with or at risk of metabolic syndrome in a disadvantaged rural area. The study findings thus contribute to the prevention and control of chronic diseases.

## Abbreviations

APAN: albany physical activity and nutrition
AUSDRISK: Australian type 2 diabetes risk assessment tool
BMI: body mass index
GEE: generalised estimating equation
HDL: high density lipoprotein
IDF: International Diabetes Federation
IPAQ-SF: international physical activity questionnaire (short form)
MET: metabolic equivalent of task
